# Medial prefrontal-thalamic white matter microstructure is associated with harm avoidance in OCD: a discovery and transdiagnostic replication study

**DOI:** 10.1038/s41386-026-02357-7

**Published:** 2026-01-31

**Authors:** João Paulo Lima Santos, Amelia Versace, Manan Arora, Michele A. Bertocci, Henry W. Chase, Simona Graur, Lisa Bonar, Chiara Maffei, Anastasia Yendiki, Christina L. Boisseau, Suzanne N. Haber, Steven A. Rasmussen, Mary L. Phillips

**Affiliations:** 1 Department of Psychiatry, University of Pittsburgh, Pittsburgh, PA USA; 2 Magnetic Resonance Research Center, University of Pittsburgh, Pittsburgh, PA USA; 3 Department of Radiology, McLean Hospital, Harvard Medical School, Boston, MA USA; 4 Department of Psychiatry and Behavioral Sciences, Northwestern University Feinberg School of Medicine, Chicago, IL USA; 5 Department of Psychiatry and Human Behavior, Warren Alpert Medical School of Brown University, Providence, RI USA; 6 Butler Hospital, Providence, RI USA; 7 Department of Psychiatry, McLean Hospital, Harvard Medical School, Boston, MA USA; 8 School of Medicine and Dentistry, University of Rochester Medical Center, Rochester, NY USA

**Keywords:** Predictive markers, Psychiatric disorders

## Abstract

Identifying neural mechanisms underlying harm avoidance and incompleteness in OCD and other psychiatric disorders is critical for improving diagnostic precision and developing targeted treatments. However, little is known about the neural pathways underlying these symptom dimensions across clinical populations. The goal of this study was to use diffusion MRI to replicate and extend prior findings in OCD to a transdiagnostic sample of healthy controls (HC) and individuals with Obsessive-Compulsive Disorder (OCD), Obsessive-Compulsive Personality Disorder (OCPD), and non-OCD disorders (e.g., anxiety, post-traumatic stress disorder). Connections between prefrontal (dorsomedial, dorsolateral, ventromedial, and ventrolateral) and subcortical regions (thalamus and striatum) were reconstructed using whole brain tractography in 38 HC (mean age [SD] = 30.97 [10.62), 47 OCD (mean age [SD] = 32.34 [12.23]), 21 OCPD (mean age [SD] = 35.67 [14.65]), and 20 Non-OCD (mean age [SD] = 35.90 [14.26]) participants. Fractional anisotropy (FA) was derived for each connection. Harm avoidance and incompleteness symptom dimensions were assessed using the Obsessive-Compulsive Trait Core Dimensions Questionnaire. In a first replication model, including individuals with OCD and HC, higher left (*β* = 0.29, *P* = 0.009, *Q* = 0.048) and right (*β* = 0.30, *P* = 0.005, *Q* = 0.0048) dorsomedial PFC-thalamus FA was associated with higher harm avoidance, with the left connection also associated with higher incompleteness (*β* = 0.29, *P* = 0.009, *Q* = 0.048). In additional models adding in OCPD and non-OCD participants, only associations among left (*β* = 0.20, *P* = 0.027, *Q* = 0.040) and right (*β* = 0.18, *P* = 0.035, *Q* = 0.045) dorsomedial PFC-thalamus FA and harm avoidance remained significant. There were no associations involving PFC-striatum connections. Dorsomedial PFC-thalamus FA was associated with harm avoidance, but less so with incompleteness. Our findings suggest that higher dorsomedial PFC-thalamic FA is associated with higher harm avoidance across diagnostic groups, providing transdiagnostic neural targets for future treatment developments.

## Introduction

Obsessive-compulsive disorder (OCD) is characterized by intrusive thoughts and compulsive behaviors, with harm avoidance and incompleteness being central symptom dimensions [1–3]. While these features are prominent characteristics of OCD, they are not exclusive to the disorder. Harm avoidance involves heightened sensitivity to potential threats and excessive avoidance behaviors and is also observed in anxiety disorders, where it drives similar avoidance patterns [1, 2, 4]. Incompleteness, characterized by a persistent sense of imperfection or the need for things to feel “just right” [1, 2], frequently occurs in OCD but is also observed in other disorders, including obsessive-compulsive personality disorder (OCPD) [5, 6]. Although harm avoidance and incompleteness are observed in other psychiatric disorders and even healthy individuals [3, 4], the understanding of the neural mechanisms underlying these symptom dimensions in OCD and other populations remains largely unknown.

Our recent neuroimaging work has begun to elucidate the shared neural mechanisms underlying harm avoidance and incompleteness in OCD and healthy individuals. Using resting state MRI, we showed an inverse relationship between medial prefrontal (PFC)/dorsal anterior cingulate cortex-amygdala connectivity and harm avoidance across OCD and healthy individuals [7]. The dorsomedial PFC plays a critical role in evaluating situational demands and preparing adaptive actions by assessing risks and guiding appropriate behavioral responses to potential threats [8, 9]. More recently [10], using diffusion MRI, we found that denser connectivity between dorsal and ventral medial PFC and subcortical regions (thalamus and striatum) was associated with greater incompleteness in both groups [10]. The ventromedial PFC integrates the emotional valence of stimuli to guide appropriate behavioral responses [11, 12] and might underlie the heightened emotional discomfort driving maladaptive behaviors in OCD [13]. Together, these findings emphasize the role of the medial PFC in these symptom dimensions across these two populations. However, it is uncertain whether these relationships extend to other psychiatric disorders. Elucidating these relationships will determine whether these associations reflect transdiagnostic mechanisms or are more specific to OCD, ultimately advancing the understanding of the transdiagnostic neural mechanisms underlying these symptom dimensions and informing the development of targeted treatments. Replication in independent samples and inclusion of individuals with anxiety and personality disorders are critical next steps.

The goals of this study were thus to replicate and extend our prior diffusion MRI findings indicating that denser connectivity between the medial PFC and subcortical regions is associated with greater symptom dimension severity [10]. Diffusion MRI enables objective quantification and reconstruction of white matter tracts and cortical-subcortical connections, offering key advantages over other MRI techniques—being able to infer microstructural properties of different brain tissues, which make it especially well-suited for replicating and extending previous white matter findings in OCD [10, 14–19]. First, we aimed to replicate our published findings [10] in a new sample of older healthy controls (HC) and OCD participants with higher levels of harm avoidance and incompleteness. Second, we aimed to extend these findings transdiagnostically by extending the new sample of HC and OCD participants to include new participants with OCPD and non-OCD disorders (participants without OCD and OCPD but showing other clinically significant disorders). Finally, we aimed to further extend the findings from this new sample by examining these connections in a combined sample from both the new and published study [10], resulting in a more diverse clinical sample with a wider range of symptoms. To achieve our goals, we reconstructed white matter connections between the PFC (dorsal/ventral, medial/lateral) and subcortical regions (thalamus and striatum) using whole-brain tractography. Tensor-based metrics, including FA—a marker of white matter collinearity—were extracted for statistical analyses, with radial diffusivity (RD) and axial diffusivity (AD) as secondary variables. Building on our previous research [10] and given that FA is positively associated with the density of fibers [20, 21], we hypothesized that higher FA in white matter connections between medial PFC and thalamus/striatum would be associated with greater harm avoidance and incompleteness in our (1) replication and (2) extended samples.

## Methods

### Participants

The study obtained approval from the Butler Hospital Institutional Review Board. Participants were recruited from community and clinical settings in and around Massachusetts and Rhode Island, as well as through targeted social media advertisements. Inclusion criteria were: (1) age 18 to 65, (2) Diagnostic and Statistical Manual of Mental Disorders, Fifth Edition (DSM-5) diagnosis of the following: OCD, OCPD, post-traumatic stress disorder, hoarding disorder, body dysmorphic disorder, and anxiety disorders (panic disorder, agoraphobia, and social anxiety disorder), (3) English speaking, and (4) willing and able to provide written informed consent. Exclusion criteria were: (1) cognitive impairment (organic brain syndrome, dementia) that would interfere with study participation, ability to provide informed consent, or completion of self-report questionnaires, (2) current psychotic disorder, (3) psychiatric medications other than serotonin reuptake inhibitors or medications taken for sleep or occasional anxiety (e.g., hydroxyzine, trazodone, etc.), (4) pre-morbid IQ < 85 as measured by the National Adult Reading Test, (5) implanted metallic substances, metallic tattoos received prior to 1990, and (6) pregnancy and any other conditions not allowed in the scanner that would represent a safety risk for participants.

In total, 188 clinical (OCD, OCPD, non-OCD) participants and 48 HC were enrolled. The non-OCD disorders group included participants without OCD or OCPD who met criteria for other clinically significant conditions, including social anxiety disorder, panic disorder, post-traumatic stress disorder, hoarding disorder, and body dysmorphic disorder (Supplementary Table 1). No participant reported major depressive disorder.

Neuroimaging data were available for 111 clinical participants and 41 HC. After quality control, 47 OCD (mean age [SD] = 32.34 [12.23] y; 19.15% female), 38 HC (mean age [SD] = 30.97 [10.62] y; 28.95% female), 21 OCPD (mean age [SD] = 35.67 [14.25] y; 23.81% female), and 20 with non-OCD disorders (mean age [SD] = 35.90 [14.26] y; 20.00% female) were included for analyses. Clinical and demographic characteristics of each sample are described in Table 1 and Supplementary Tables 2–3. See Supplementary Table 3 and Lima Santos et al. [10] for clinical and demographic characteristics of the original OCD participant and HC sample.

**Table 1:** Clinical and demographic characteristics of the current samples.

Variable	Sample 1		Sample 2	Sample 3
	HC (*N* = 38)	OCD (*N* = 47)^a^	OCPD (*N* = 21)^a^	Non-OCD disorders (*N* = 20)^a^
Age (years), mean [SD]	30.97 [10.62]	32.34 [12.23]	35.67 [14.25]	35.90 [14.26]
Sex at birth				
Female, *N* (%)	27 (71.05%)	38 (80.85%)	16 (76.19%)	16 (80%)
Male, *N* (%)	11 (28.95%)	9 (19.15%)	5 (23.81%)	4 (20%)
Education level				
Lower, *N* (%)	27 (71.05%)	39 (82.98%)	18 (85.71%)	14 (70%)
Higher, *N* (%)	11 (28.95%)	8 (17.02%)	3 (14.29%)	6 (30%)
Harm avoidance^b^, mean [SD]	1.84 [3.63]	24.55 [8.80]	16.76 [6.66]	11.3 [9.07]
Incompleteness^b^, mean [SD]	2.79 [4.66]	24.64 [9.21]	19.95 [6.25]	10.9 [9.94]
YBOCS, mean [SD]	-	21.17 [4.48]	10.29 [6.66]	5.95 [5.72]
Psychotropic medications, *N* (%) ^c^	0 (0.00%)	29 (61.7%)	8 (38.1%)	11 (55%)
Selective serotonin reuptake inhibitors (SSRIs), *N* (%)	0 (0.00%)	24 (51.06%)	8 (38.1%)	11 (55%)
OCD illness duration (years), mean [SD]	-	15.67 [11.81]	-	-

*HC* Healthy controls, *OCD* Obsessive-compulsive disorder, *OCPD* Obsessive-compulsive personality disorder, *YBOCS* Yale-Brown Obsessive Compulsive Disorder Scale.

^a^Distribution of comorbidities in each group is described in the Supplements (Supplementary Table 3).

^b^OCD participants in the current sample showed significantly higher harm avoidance (*F* = 4.00, *P* = 0.048) and incompleteness (*F* = 5.12, *P* = 0.026) than the original sample. Details about the original sample are provided in Supplementary Table 2.

^c^Five participants in the OCD group from sample 1 reported benzodiazepines.

### Assessments

#### Diagnostic assessment

Clinical diagnoses were established by trained interviewers using the Anxiety and Related Disorders Interview Schedule for DSM-5 [22], OCPD module of the Structured Clinical Interview for DSM-5 Personality Disorders (SCID-5-PD) [23], and the Hoarding Rating Scale-Interview (HRS-I) [24]. See Supplementary methods for additional details.

#### Symptom dimension measures

The severity of harm avoidance and incompleteness symptoms dimension was assessed using the Obsessive-Compulsive Trait Core Dimensions Questionnaire (OC-TCDQ) [25].

#### Other measures

Demographic and clinical data were collected for all participants, including age, sex, educational level, and psychotropic medication (Selective Serotonin Reuptake Inhibitors and benzodiazepines) use at the time of the scan. Educational level was categorized into lower or higher than a college degree. Depressive symptoms were characterized using the Beck Depression Inventory [26]. For OCD participants, illness duration was calculated based on the age of OCD onset (collected with the ADIS-5) [22]. Additionally, the Yale-Brown Obsessive Compulsive Scale [27] assessed OCD symptom severity in participants with psychiatric disorders.

### Neuroimaging

The protocols of the new and original [10] studies are described in the Supplemental Material. As reported in the original study [10], four steps were performed to extract metrics from the connections between PFC and subcortical regions: 1. whole brain tractography; 2. cortical parcellation and subcortical segmentation of regions of interest (ROI); 3. extraction of PFC-thalamus and PFC-striatum connections; and 4. diffusion metrics.

#### Whole-brain tractography

After preprocessing diffusion MRI data for echo-planar imaging distortion, eddy current and subject motion [28, 29], Mrtrix3 [30] was used for whole brain tractography in native space using random white matter seeds (*N* = 50 random seeds). White matter streamlines were then warped into Montreal Neurological Institute (MNI) space.

#### Cortical parcellation and subcortical segmentation of ROIs

Cortical and subcortical brain regions were mapped using Freesurfer 7.4.1 [31]. We selected two binary masks for subcortical regions: thalamus and striatum (a combination of caudate and putamen Freesurfer masks). For the PFC, we collapsed cortical regions into four ROIs in each hemisphere: (1) ventral lateral PFC, combining the pars orbitalis, pars triangularis, and pars opercularis; (2) ventral medial PFC, combining the medial orbitofrontal cortex and gyrus rectus; (3) dorsal lateral PFC, combining the rostral and caudal middle frontal regions; and (4) dorsal medial PFC, including the caudal anterior cingulate cortex. For all participants, binary masks were warped into MNI space.

#### Extraction of PFC connections

Based on the whole-brain tractography, we extracted the streamlines connecting cortical and subcortical ROIs. The number of streamlines refers to the number of reconstructed streamlines within our ROI. Quality assurance determined the usability of data (see Supplemental Materials for more details).

#### Additional connections

In the cortical ROIs described in 2.3.2, the superior frontal gyrus (SFG) was excluded due to the lack of a medial/lateral parcellation necessary to accurately capture its contribution to the dorsomedial and dorsolateral PFC. However, connections between the SFG and the thalamus or striatum were also reconstructed to explore the potential involvement of these connections in the symptom dimensions under study.

#### Diffusion metrics

FA, RD, and AD maps were derived for each participant in native space and were then registered to MNI space. Mean metrics were extracted for each bundle of cortical-subcortical streamlines. In addition, the number of cortical-subcortical streamlines of each connection was extracted and used as a covariate in the statistical analyses. To account for the acquisition differences between new and original samples (e.g., scanner, MRI protocol), we used COMBAT [32] to harmonize the data. In our previous work [10], we used a multi-shell diffusion imaging protocol, which enabled the use of Neurite Orientation Dispersion and Density Imaging (NODDI) measurements [33–35]. In the current study, we use FA because tensor-based models are suitable for single-shell diffusion imaging protocols. Although FA is less specific than NODDI, it remains a widely accepted and valid approach for assessing white matter microstructure, particularly in large-scale or harmonized datasets.

### Statistical analyses

In all models, age, sex, educational level, and total number of streamlines were included as covariates. FDR was used to account for multiple comparisons (*Q* ≤ 0.050) [36]. In addition, given that the distribution of OCD symptom dimensions across participants was not normally distributed, these scores were log-transformed before statistical analyses. Model comparison using AIC showed that linear models outperformed the non-parametric alternative (Supplementary Table 4).

#### Hypothesis 1: replication model

We performed multivariate regression models to identify relationships between FA extracted from the cortical-subcortical connections and OCD symptom dimensions (harm avoidance or incompleteness) in *Sample 1* (38 and 47 OCD participants). In these analyses, FA was the independent variable, and OCD symptom dimensions, one at a time, were the dependent variables.

#### Hypothesis 2: extended models

Multivariate regression models investigated whether the significant FA associations identified in *2.4.1* were also present after including other clinical participants. These models were performed in the following samples:

i. Extended sample 1: adding OCPD (*N* = 21) to the replication sample.
ii. Extended sample 2: adding OCPD (*N* = 21) and non-OCD (*N* = 20) to the replication sample.
iii. Extended sample 3: combing the current sample (HC = 38, OCD = 47, OCDP = 21, and Non-OCD = 20) and the original sample (HC = 42 and OCD = 44).

#### Exploratory analyses

##### Within-group models

Analyses mirroring the main models explored whether the significant findings linking OCD symptom dimensions and FA were present within each group (HC, OCD, OCPD, and Non-OCD).

##### Non-significant models in the replication analyses

Models explored whether the non-significant connections in *2.4.1* analyses were associated with symptom dimensions in the extended (*2.4.2*) models.

##### Other tensor-based metrics

As described in *2.4.1*, multivariate regression models evaluated the association between other tensor-based metrics (AD or RD) and OCD dimensions (harm avoidance or incompleteness) in Sample 1.

##### Psychotropic medication

Analysis of covariance (ANCOVA) evaluated the effect of taking psychotropic medication (yes/no) on symptom dimensions (harm avoidance and incompleteness) in OCD participants in Sample 1. In addition, ANCOVAs evaluated the effect of psychotropic medications on FA metrics of all cortical-subcortical connections.

##### Illness duration

Analyses evaluated the effect of illness duration (difference in time between age at scan and age of onset) on tensor-based metrics (FA) in the OCD samples.

##### Additional connections

As described in *2.4.1* analyses, exploratory models evaluated whether FA of SFG-thalamus or -striatum connections were associated with OCD symptom dimensions (harm avoidance and incompleteness).

##### Additional between-group models

ANCOVA models investigated between-group differences in the number of streamlines and FA of the reconstructed connections between the thalamus or striatum and PFC regions. Additionally, ANCOVA models investigated group differences in FA between HC and all clinical groups combined (OCD, OCPD, and Non-OCD).

##### Depressive symptoms

Analyses evaluated whether FA of thalamic and striatal connections was associated with depressive symptoms in the new sample with HC, OCD, OCDP, and Non-OCD participants.

## Results

### Hypothesis 1: replication model

**Fig. 1:**
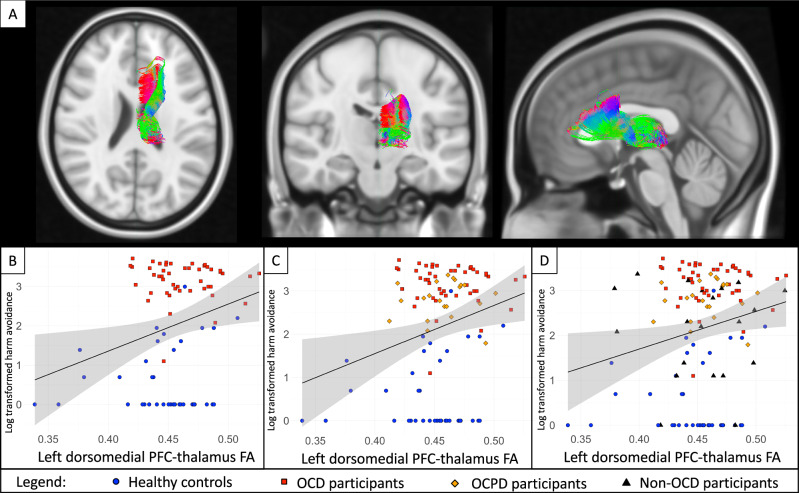
Relationship between FA of the left dorsomedial PFC-thalamus and harm avoidance in the current sample. **A** shows the reconstructed connection between left dorsomedial PFC and thalamus. Axial, coronal, and sagittal slices are shown. Colors indicate streamline orientation: green (anterior-posterior), blue (superior-inferior), and red (right-left). In this panel, the background is the standard Montreal Neurological Institute 152 space 1-mm brain. In **B**–**D** the x-axis shows the left dorsomedial PFC-thalamus FA and the y-axis shows the log-transformed harm avoidance. Log transformation was performed as log(score + 1) to include participants with a score of 0 and avoid producing negative infinity. In **B** healthy controls and OCD participants are displayed. In **C** healthy controls, OCD, and OCPD participants are displayed. In **D** healthy controls, OCD, OCPD, and non-OCD participants are displayed. In all panels, the gray area represents 95% confidence intervals, and the black line represents the regression line. FA Fractional anisotropy, PFC Prefrontal cortex, OCD Obsessive-compulsive disorder, OCPD Obsessive-Compulsive Personality Disorder.

Models revealed that higher FA in the left (Fig. 1) and right (Supplementary Fig. 1) dorsomedial PFC-thalamus connections were associated with higher harm avoidance (Table 2). In addition, higher FA in the left dorsomedial PFC-thalamus connection was associated with higher incompleteness (Table 2 and Supplementary Fig. 2; all *Q* < 0.050). All three associations showed medium effect sizes (Table 2). There were no associations between PFC-striatum connections and OCD symptom dimensions (Supplementary Table 5).

**Table 2:** Relationships between FA of PFC-thalamus connections and OCD symptom dimensions in the replication model (HC and OCD).

OCD symptom-dimensions	Hemisphere	Tract	*β*	*P*^a^	*η*^2 b^	Q^a,c^
Harm avoidance	Left	Dorsolateral PFC	0.12	0.311	0.02	0.829
		Dorsomedial PFC	0.29	**0.009**	0.09	**0.048**
		Ventrolateral PFC	−0.01	0.939	<0.01	0.984
		Ventromedial PFC	−0.01	0.926	<0.01	0.984
	Right	Dorsolateral PFC	−0.02	0.868	0.00	0.984
		Dorsomedial PFC	0.30	**0.005**	0.06	**0.048**
		Ventrolateral PFC	0.08	0.469	0.01	0.938
		Ventromedial PFC	−0.07	0.571	0.01	0.984
Incompleteness	Left	Dorsolateral PFC	0.05	0.690	<0.01	0.984
		Dorsomedial PFC	0.29	**0.009**	0.09	**0.048**
		Ventrolateral PFC	0.05	0.667	0.01	0.984
		Ventromedial PFC	0.00	0.984	<0.01	0.984
	Right	Dorsolateral PFC	0.03	0.802	<0.01	0.984
		Dorsomedial PFC	0.28	**0.013**	0.03	*0.052*
		Ventrolateral PFC	0.09	0.387	0.02	0.885
		Ventromedial PFC	−0.16	0.183	0.03	0.586

*HC* Healthy controls, *FA* Fractional Anisotropy, *OCD* Obsessive-compulsive disorder.

^a^*P*-values ≤ 0.05 are reported in bold characters; *P* values between 0.05 and 0.10 are reported in italics.

^b^*η*^2^ effect size benchmarks: negligible (*η*^2^ ≥ 0.01), small (0.01 ≤ *η*^2^ < 0.06), medium (0.06 ≤ *η*^2^ < 0.14), and large (*η*^2^ ≥ 0.14) partial effects sizes [45].

^c^ Q represents the *P*-values after correction for multiple comparisons.

### Hypothesis 2: extended models

#### Extended sample 1 (HC, OCD, and OCPD participants)

Higher FA in the left (Fig. 1) and right (Supplementary Fig. 1) dorsomedial PFC-thalamus connections was associated with higher harm avoidance, and higher FA in the left dorsomedial PFC-thalamus (Supplementary Fig. 2) connection was associated with higher incompleteness (Table 3; all *Q* < 0.050)

**Table 3:** Extended models investigating the relationships between FA of PFC-thalamus connections and OCD symptom dimensions.

Models	OCD symptom-dimension	Tract	*β*	*P*^a^	*η*^2 b^	Q^a,c,d^
Extended sample 1^e^	Harm avoidance	Left dorsomedial PFC	0.24	**0.015**	0.08	**0.036**
		Right dorsomedial PFC	0.22	**0.020**	0.03	**0.036**
	Incompleteness	Left dorsomedial PFC	0.25	**0.011**	0.08	**0.036**
Extended sample 2^f^	Harm avoidance	Left dorsomedial PFC	0.20	**0.027**	0.05	**0.040**
		Right dorsomedial PFC	0.18	**0.035**	0.02	**0.045**
	Incompleteness	Left dorsomedial PFC	0.13	0.167	0.03	0.167
Extended sample 3^g^	Harm avoidance	Left dorsomedial PFC	0.19	**0.008**	0.04	**0.036**
		Right dorsomedial PFC	0.11	0.112	0.01	0.126
	Incompleteness	Left dorsomedial PFC	0.17	**0.018**	0.03	**0.036**

*FA* Fractional Anisotropy, *OCD* Obsessive-compulsive disorder.

^a^*P* ≤ 0.05 are reported in bold characters; *P* values between 0.05 and 0.10 are reported in italics.

^b^*η*^2^ effect size benchmarks: negligible (*η*^2^ ≥ 0.01), small (0.01 ≤ *η*^2^ < 0.06), medium (0.06 ≤ *η*^2^ < 0.14), and large (*η*^2^ ≥ 0.14) partial effects sizes [45].

^c^Q represents the *P*-values after correction for multiple comparison.

^d^Model fit comparisons (using AIC) for all analyses in Tables 2–3 showed that linear regression models using log-transformed outcomes consistently outperformed negative binomial models (see Supplementary Table 4). For significant findings in Table 2 and all findings in Table 3 (which used linear regression models), the use of negative binomial models produced similar findings. We found significant findings in the (Table 2) replication sample (*P* ≤ 0.017, *Q* ≤ 0.017) and extended sample 1 (*P* ≤ 0.048, *Q* ≤ 0.048). In extended samples 2 (*P* ≤ 0.081, *Q* ≤ 0.122) and 3 (*P* ≤ 0.046, *Q* ≤ 0.069), negative binomial models yielded near-significant effects. FDR corrections were applied across all three models within each sample.

^e^Extended sample 1 includes participants from sample 1 (healthy controls and obsessive-compulsive disorder) and sample 2 (obsessive-compulsive personality disorder).

^f^Extended sample 2 includes participants from sample 1 (healthy controls and obsessive-compulsive disorder), sample 2 (obsessive-compulsive personality disorder), and sample 3 (non-OCD disorders).

^g^Extended sample 3 includes participants from sample 1 (healthy controls and obsessive-compulsive disorder), sample 2 (obsessive-compulsive personality disorder), sample 3 (non-OCD disorders), and original sample (healthy controls and obsessive-compulsive disorder).

#### Extended sample 2 (HC, OCD, OCPD, and Non-OCD participants)

Higher FA in the left (Fig. 1) and right (Supplementary Fig. 1) dorsomedial PFC-thalamus connections were associated with higher harm avoidance (Table 3; all *Q* < 0.050). There were no associations with incompleteness (Table 3).

#### Extended sample 3 (current and original samples)

**Fig. 2:**
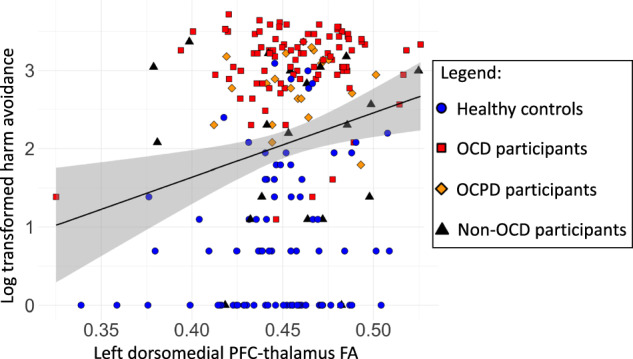
Relationship between FA of the left dorsomedial PFC-thalamus and harm avoidance in the model combining current and original samples. The x-axis shows the left dorsomedial PFC-thalamus FA and the y-axis shows the log transformed harm avoidance. Log transformation was performed as log(score + 1) to include participants with a score of 0 and avoid producing negative infinity. In this scatter plot, healthy controls, OCD, OCPD, and non-OCD participants are displayed. Healthy controls and OCD participants include the current and original samples. The gray area represents 95% confidence interval, and the black line represents the regression line. FA Fractional anisotropy, PFC Prefrontal cortex, OCD Obsessive-compulsive disorder, OCPD Obsessive-Compulsive Personality Disorder.

After including all participants (current and original samples), higher FA in the left dorsomedial PFC-thalamus connection was associated with higher harm avoidance (Fig. 2) and incompleteness (Supplementary Fig. 3; all *Q* < 0.050). There was no association between symptom dimension severity and FA in the right dorsomedial PFC-thalamus connection (Table 3).

### Exploratory analyses

#### Within-group models

There were no within-group associations between OCD symptom dimensions and FA (Supplementary Table 6).

#### Non-significant findings in the replication analyses

There was no association between non-significant findings in 2.4.1 with symptom dimensions in the extended samples (Supplementary Table 7).

#### Other tensor-based metrics

There was no association between AD or RD of PFC-thalamus or PFC-striatum with symptom dimensions (Supplementary Table 8–9).

#### Psychotropic medication

There was no effect of psychotropic medications (Supplementary Table 10).

#### Illness duration

There was no association between illness duration and FA (Supplementary Table 11).

#### Additional connections

There was no association between FA of the SFG-thalamus or -striatum connections and symptom dimensions after FDR correction (Supplementary Table 12).

#### Additional between-group models

We observed a significant between-group difference in the number of streamlines connecting the right dorsomedial PFC to the thalamus (Supplementary Table 13). No other streamline differences reached significance (all *p* > 0.05). Regarding FA, OCD participants showed significantly higher FA than HC in the left dorsomedial PFC–thalamus connection. In addition, all clinical groups combined also showed higher FA than HC in the left dorsomedial PFC–thalamus connection; however, these effects did not survive correction for multiple comparisons (Supplementary Tables 14–15).

#### Depressive symptoms

There was no association between FA and depressive symptoms (Supplementary Table 16).

## Discussion

The current study aimed to replicate previously published findings [10] in a new, independent external sample. We examined the relationship between FA in PFC-subcortical white matter connections and symptom dimensions of harm avoidance and incompleteness in HC and clinical participants, including individuals with OCD, OCPD, and non-OCD disorders. Our findings showed that higher FA in both the left and right connections between the caudal anterior cingulate cortex, represented as the dorsomedial PFC, and the thalamus was associated with greater harm avoidance, while higher FA in the left dorsomedial PFC-thalamus connection was also linked to higher incompleteness. The most consistent association across all groups was between higher FA in the left dorsomedial PFC-thalamus and greater harm avoidance. However, the remaining associations varied depending on the inclusion of non-OCD participants or a more heterogeneous OCD sample. Finally, no significant associations were observed between PFC-striatum connections and OCD symptom dimensions, nor were there any associations between other tensor-based metrics (AD and RD) and these symptom dimensions. Furthermore, psychotropic medication and illness duration did not influence these relationships.

The findings linking higher FA with greater symptom dimension severity are consistent with our previous work showing that denser connections were associated with greater symptom severity [10]. In the current sample, FA reflects the collinearity and structural coherence of white matter fibers, with higher FA suggesting denser connectivity between brain regions [20, 21]. The dorsomedial PFC plays a critical role in regulating behavior by assessing risk and guiding adaptive responses to potential threats [37–39]. In this context, increased FA in the dorsomedial PFC-thalamus connection may reflect heightened sensitivity to perceived threats, contributing to more pronounced avoidance behaviors. While our dorsomedial PFC findings were more strongly associated with harm avoidance, we also observed an association with incompleteness, where increased FA may reflect an overactive error monitoring system, driving persistent doubts or uncertainty [37–39]. Together, our findings suggest that the overall structure of the dorsomedial PFC-thalamus connection, as reflected by FA, is not exclusive to a single symptom dimension but contributes to both harm avoidance and incompleteness in OCD. These findings align with prior studies demonstrating that neuromodulation techniques, such as transcranial magnetic stimulation, can alleviate overall OCD symptom severity by reducing dorsomedial hyperconnectivity in treatment-resistant patients [40].

The association between higher FA in the left dorsomedial PFC-thalamus connection and greater harm avoidance remained significant in the models that included other clinical populations. This consistency across all models suggests that this connection might reflect a core neural mechanism for harm avoidance, independent of diagnosis, underscoring a shared neural substrate for maladaptive behavioral tendencies across diverse populations. Importantly, exploratory within-group analyses revealed no significant associations between OCD symptom dimensions and white matter measures in any group. However, group comparisons showed higher FA in the left dorsomedial-thalamus connection in OCD *versus* HC and also in all clinical groups *versus* HC, although these findings did not survive FDR correction. These findings indicate that the observed associations among OCD-related symptom dimension severity and FA in the left dorsomedial-thalamus connection are partially, but not totally, accounted for by categorical diagnostic effects. While the left hemisphere-centered nature of these findings is difficult to explain, this may reflect the suggested role of the left hemisphere in local rather than global processing [41], leading to the heightened sensitivity to local stimulus features that is associated with harm avoidance and incompleteness [1–6], and to the relative deficits in global versus local stimulus processing that have previously reported in OCD [42]. Future studies can examine this further.

The association between higher FA in the right dorsomedial PFC-thalamus connection and harm avoidance was not observed when we expanded the range of symptom dimensions by including the original sample. Given the significant difference in harm avoidance between the current and original study OCD participants (Table 1), this suggests that the right dorsomedial PFC-thalamus connection might be less robust or more sensitive to illness severity, potentially reflecting a weaker or context-dependent role in harm avoidance. Similarly, the relationship between higher FA in the left dorsomedial PFC-thalamus connection and incompleteness was no longer significant when the more heterogeneous group (non-OCD) was added to the model, suggesting that this connection might be more specific to OCD-related mechanisms, as suggested above. Notably, this association re-emerged when we expanded the symptom range within the clinical sample, indicating that the left dorsomedial PFC-thalamus connection might play a stronger role in incompleteness within OCD populations that exhibit greater symptom diversity. Together, these findings highlight the selective contributions of the dorsomedial PFC-thalamus connections to harm avoidance and incompleteness and underscore the importance of considering sample composition when interpreting these neural relationships.

In our previous work [10] using NODDI, we demonstrated that higher neurite density (NDI) in the left dorsomedial PFC-thalamus connection was associated with greater incompleteness. The current study replicated this finding using FA instead of NDI, demonstrating a medium effect size and underscoring the critical role of this connection in OCD-related neural mechanisms. Importantly, by replicating and extending these results, we provide further evidence that the dorsomedial PFC-thalamus connection might play a central role in a core dimension of OCD symptomatology. This consistent finding across methodologies strengthens confidence in the robustness of this association while offering novel insights into the broader symptom range and diagnostic heterogeneity explored in the present study. However, unlike our previous work, we did not observe any significant findings involving PFC-striatum connections in the current study. This discrepancy might result from the distinct properties captured by each diffusion MRI metric. While FA reflects the overall microstructure of white matter connections [20, 21], NODDI-derived measures such as NDI and ODI provide more nuanced information [33, 34], which might be particularly relevant for complex connections such as corticostriatal connections [43, 44]. These connections might exhibit microstructural features not fully captured by FA, but better characterized by NODDI metrics, which could help explain the absence of findings regarding PFC-striatum connections in the current study.

Additionally, in our previous work, we showed a relationship between lower ventromedial PFC-striatal FA and greater incompleteness severity. The lack of significant ventromedial PFC-related findings in the current study might be related to the severity of symptoms in the present sample. OCD participants in the current study showed significantly greater harm avoidance and incompleteness severity than the original sample in our previous work (Table 1). It is therefore plausible that ventromedial PFC-related connections might play a more pivotal role in the pathophysiology of lower severity OCD symptoms. Conversely, alterations in FA in the dorsomedial PFC-thalamus connection might become more prominent in individuals with more severe symptoms, suggesting an illness-staging effect, where there is a shift from alterations in ventromedial to dorsomedial PFC-related connections with increasing severity of OCD-related psychopathology. Future work incorporating both FA and NODDI metrics across diverse samples is needed to help clarify the specific contributions and the relative sensitivity of different diffusion MRI metrics in detecting microstructural alteration associated with OCD-related psychopathology.

While this study builds on prior work, several limitations should be noted. We used automatic labeling of brain regions, which may not capture individual anatomical differences. Future studies should consider combining individualized and automatic labeling approaches to better characterize the strengths and limitations of each method. The single-shell diffusion MRI limited our ability to apply advanced techniques such as NODDI, and future multi-shell approaches could provide more detailed insights into white matter microstructure, including specific segments of the dorsomedial PFC–thalamic tract. Although the lack of significant findings in the within-group models supports our transdiagnostic interpretation, larger studies with greater variability in symptom severity are needed to address limited statistical power in small groups. While we found no effects of medications taken at the time of scanning, medication use plays an important role in the neurobiology of OCD, and future studies should examine how changes in medications over time impact these connections. Our replication analysis was conceptual rather than direct due to differences in scanner, sequence, and sample, but overlapping findings support the robustness of the results. The sample size, though typical for OCD neuroimaging studies, was relatively small, and larger, more diverse samples are needed. The study was not preregistered, and future work could preregister analyses and adopt individualized brain region mapping to improve precision.

In conclusion, this study contributes to a deeper understanding of the neural mechanisms underlying OCD symptom dimensions by highlighting the role of the connection between the dorsomedial PFC, more specifically the caudal anterior cingulate cortex, to the thalamus in harm avoidance and incompleteness. The consistent involvement of the left dorsomedial PFC-thalamus connection with harm avoidance across all groups underscores the possibility of a shared neural substrate for maladaptive behavioral tendencies across diverse populations. This shared connection is particularly important for understanding the common neural mechanisms underlying these symptom dimensions, although these findings should be interpreted with caution, given the modest sample size and the observational nature of the study. Rather than suggesting immediate treatment targets, our results provide a preliminary step toward identifying candidate neural pathways that may be explored in future research. Future studies should continue to explore these connections across clinical populations and symptom domains, with the aim of informing more targeted and effective treatment strategies that address these core neural mechanisms.

## Supplementary information

Supplemental Materials

## Data Availability

The data that support the findings of this study are not publicly available due to privacy and ethical restrictions. Data may be made available from the Principal Investigators upon reasonable request and with approval from the relevant ethics board.
