# Supplementary material for: Medial prefrontal-thalamic white matter microstructure is associated with harm avoidance in OCD: a discovery and transdiagnostic replication study
Source: Neuropsychopharmacology. 2026 Jan 31;51(8):1424–31. doi: 10.1038/s41386-026-02357-7 (PMC13291171; doi:10.1038/s41386-026-02357-7)
Supplement: Supplementary file 1 — Supplemental Materials [file 41386_2026_2357_MOESM1_ESM.docx]

**SUPPLEMENTAL MATERIALS**

**Supplemental Methods.**

*Assessments*.

The Anxiety and Related Disorders Interview Schedule for DSM-5[1] was used to ascertain reliable diagnoses of anxiety disorders (social anxiety disorder, panic disorder, agoraphobia) and obsessive-compulsive disorder. The OCPD module of the Structured Clinical Interview for DSM-5 Personality Disorders (SCID-5-PD)[2] was used to establish diagnosis of OCPD and the Hoarding Rating Scale-Interview (HRS-I)[3], was used to assess hoarding disorder. All interviewers had a least a bachelor’s degree and were trained to reliability by MD and PhD level experts in OCD research and treatment. Interviewers completed a series of observational, didactic, and participatory training activities including conducting assessments with feedback from experienced interviewers and audiotaping for inter-rater reliability calculations. New interviewers were required to achieve excellent inter-rater reliability with doctoral level trainers and senior raters (interclass correlation coefficient > 0.85 for anxiety, obsessive-compulsive and related diagnoses as well as YBOCS total score). Throughout the study, fidelity to training was monitored via weekly meetings to review interviews as well as periodic, random calculation of inter-rater reliability statistics.

*Neuroimaging*[4].

*Protocol - New study.* Images were acquired on a 3T Siemens Prisma at the Brown’s MRI facility with a 64-channel headcoil. MPRAGE structural (176 slices, voxel size=1.0x1.0x1.0mm3, Interleaved, TR 2530ms Flip angle 7.0 degrees, GRAPPA acceleration factor 2, TE1=1.64ms, TE2=3.5ms, TE3=5.36ms, TE4=7.22ms; duration=6’03’’) and multi-band accelerated EPI sequence developed at University of Minnesota's Center for Magnetic Resonance Research (CMRR; TR = 17,000ms, TE=78ms, FOV= 24cm, 128 Acquisition Matrix with slice thickness=2mm resulting in isotropic 2x2x2mm3 voxels, MB factor 4, PED=P>>A; monopolar, including 60 noncollinear diffusion directions with b=700s/mm2 and 4 baseline scans with b=0s/mm2; duration=13’22’’) were acquired.

*Protocol - Original study.* Images were acquired on a 3T Siemens Magnetom Prisma at the Magnetic Resonance Research Center, University of Pittsburgh Medical Center Health System, USA. A built-in whole-body coil and a 32-channel head coil were used for the RF transmission and the RF reception, respectively. MPRAGE structural (176 slices, voxel size=1.0x1.0x1.0mm3, Interleaved, TR 1520ms Flip angle 8.0 degrees, TE=3.17ms; duration=7’20’’) was collected. A single-shot spin-echo echo planar imaging (SE-EPI) sequence was acquired with 197 optimized non-colinear diffusion-weighting gradient directions (32 volumes with b= 700 s/mm2, 65 volumes with b= 1000 s/mm^2^, 100 volumes with b= 2500 s/mm^2^) and 13 reference volumes with b=0 s/mm^2^ (repetition time (TR)=3000ms, echo time (TE)=120ms, flip angle=90, field-of-view (FOV)=256×256, 2x2x2mm^3^ isotropic voxel, SMS factor=4, acquisition time=630 seconds). In accordance with a forward-reverse protocol, the acquisition was collected twice with opposite phase encoding directions 4(P>>A and A>>P). The sampling scheme was based on the strength and number of gradient directions for each shell to ensure optimal angular coverage across the entire sequence.[5] Diffusion-weighted images were corrected for eddy current, subject motion and EPI distortion using topup and eddy, within FMRIB’s Software Library (FSL).[6-9]

*Quality control.* Quality control of reconstructed tracts involved evaluating the presence, overall appearance, and continuity of streamlines. The presence of streamlines was assessed based on the number of streamlines connecting each pair of regions of interest, with connections classified as failed if no streamlines (i.e., zero) were detected. In the final sample, the number of streamlines between the Thalamus and PFC ranged as follows: dorsomedial (Left: 23-6,691; Right: 3,016-3,238), dorsolateral (Left: 1,547-26,101; Right: 2,975-25,489), ventromedial (Left: 400-27,638; Right: 384-10,230), and ventrolateral (Left: 195-17,436; Right: 140-19,627). For the Striatum–PFC tracts, the corresponding ranges were: dorsomedial (Left: 29-4,304; Right: 61-4,711), dorsolateral (Left: 4,820-45,615; Right: 3,154-44,486), ventromedial (Left: 2,127-29,118; Right: 838-15,990), and ventrolateral (Left: 1,047-28,007; Right: 2,204-25,680). Overall appearance and continuity were assessed through visual inspection to confirm anatomically plausible trajectories and to ensure that reconstructed streamlines were continuous between regions of interest, with connections classified as failed if these criteria were not met.

**SUPPLEMENTAL TABLES**

**Supplemental Table 1. Distribution of psychiatric disorders in the sample.**

| Comorbidity | OCD (N=47) ^a^ | OCPD (N=21) ^a^ | Non-OCD disorders (N=20) ^a^ |
| --- | --- | --- | --- |
| OCPD | 18 | 21 | 0 |
| Social anxiety disorder | 29 | 14 | 16 |
| Panic disorder | 7 | 3 | 2 |
| Agoraphobia | 7 | 1 | 0 |
| Post-traumatic stress disorder | 6 | 4 | 1 |
| Hoarding disorder | 10 | 7 | 3 |
| Body dysmorphic disorder | 0 | 0 | 2 |

Abbreviations: OCD, Obsessive-compulsive disorder; OCPD, Obsessive-compulsive personality disorder

^a^ Some participants had more than one comorbidity, so these counts are not mutually exclusive.

**Supplemental Table 2. Between-group differences in clinical and demographic characteristics of the current samples.**

| **Variable** | Sample 1 HC vs. Sample 1 OCD | | Sample 1 HC vs. Sample 2 OCPD | | Sample 1 HC vs. Sample 3 non-OCD disorders ^a^ | |
| --- | --- | --- | --- | --- | --- | --- |
|  | t(83) or x2 | P ^b^ | t(57) or x2 | P ^b^ | t(56) or x2 | P ^b^ |
| Age | -0.54 | 0.589 | -1.44 | 0.156 | -1.49 | 0.142 |
| Sex at birth | 0.64 | 0.423 | 0.01 | 0.905 | 7.20 | **0.007** |
| Education | 1.10 | 0.294 | 0.90 | 0.343 | 3.20 | *0.074* |
| Harm avoidance | -17.47 | **< 0.001** | -11.03 | **< 0.001** | -5.91 | **< 0.001** |
| Incompleteness | -14.31 | **< 0.001** | -9.68 | **< 0.001** | -4.26 | **< 0.001** |

Abbreviations: HC, Healthy controls; OCD, Obsessive-compulsive disorder; OCPD, Obsessive-compulsive personality disorder

^a^ Distribution of non-OCD psychiatric disorders: Social anxiety, N=14; Social anxiety and post-traumatic stress disorder, N=1; Social anxiety and Hoarding disorder, N=1; Hoarding disorder, N=2; Panic disorder, N=2.

^b^ P-values ≤ 0.05 are reported in bold characters; P values between 0.05 and 0.10 are reported in italics.

**Supplemental Table 3. Clinical and demographic characteristics of the original sample.**

| **Variable** | **HC (N=44)** | **OCD (N=42)** | t(84) or x2 | P ^a^ |
| --- | --- | --- | --- | --- |
| Age (years), mean [SD] | 23.74 [4.15] | 23.56 [4.4] | 0.20 | 0.845 |
| Sex at birth |  |  | 0.08 | 0.774 |
| Female, N (%) | 27 (61.36%) | 28 (66.67%) |  |  |
| Male, N (%) | 17 (38.64%) | 14 (33.33%) |  |  |
| Education level |  |  |  |  |
| Lower, N (%) | 23 (52.27%) | 24 (57.14%) | x2 = 0.06 | 0.813 |
| Higher, N (%) | 21 (47.73%) | 18 (42.86%) |  |  |
| Harm avoidance, mean [SD] | 3.25 [5.08] | 21.07 [7.57] | -12.47 | **< 0.001** |
| Incompleteness, mean [SD] | 4.43 [5.04] | 20.21 [9.21] | -9.02 | **< 0.001** |
| YBOCS, mean [SD] | - | 20.62 [3.47] | - | |
| Psychotropic medications, N (%) | - | 17 (40.48%) | - | |
| Selective serotonin reuptake inhibitors (SSRIs), N (%) | - | 17 (40.48%) | - | |
| OCD illness duration (years), mean [SD] | - | 14.4 [8.18] | - | |

^a^ P-values ≤ 0.05 are reported in bold characters.

**Supplemental Table 4. Model fit comparisons.**

| Relationships between FA of PFC-thalamus connections and OCD symptom-dimensions in the replication model (HC and OCD). | | | | |
| --- | --- | --- | --- | --- |
| OCD symptom-dimensions | Hemisphere | Tract | AIC^a^ | |
|  |  |  | Linear regression models | Negative binomial models |
| Harm avoidance | Left | Dorsolateral PFC | **241.14** | 308.70 |
|  |  | Dorsomedial PFC | **233.71** | 303.37 |
|  |  | Ventrolateral PFC | **241.81** | 309.43 |
|  |  | Ventromedial PFC | **241.01** | 308.33 |
|  | Right | Dorsolateral PFC | **236.18** | 305.39 |
|  |  | Dorsomedial PFC | **229.85** | 299.88 |
|  |  | Ventrolateral PFC | **241.32** | 308.93 |
|  |  | Ventromedial PFC | **242.12** | 309.55 |
| Incompleteness | Left | Dorsolateral PFC | **247.65** | 313.83 |
|  |  | Dorsomedial PFC | **238.34** | 306.95 |
|  |  | Ventrolateral PFC | **247.82** | 314.14 |
|  |  | Ventromedial PFC | **246.34** | 312.27 |
|  | Right | Dorsolateral PFC | **244.52** | 311.63 |
|  |  | Dorsomedial PFC | **236.43** | 305.22 |
|  |  | Ventrolateral PFC | **247.23** | 313.61 |
|  |  | Ventromedial PFC | **245.97** | 312.56 |
| Extended sample 1 ^b^ | | | | |
| OCD symptom-dimensions | Hemisphere | Tract | AIC^a^ | |
|  |  |  | Linear regression models | Negative binomial models |
| Harm avoidance | Left | Dorsomedial PFC | **291.90** | 373.28 |
|  | Right | Dorsomedial PFC | **289.34** | 371.80 |
| Incompleteness | Left | Dorsomedial PFC | **298.33** | 379.46 |
| Extended sample 2 ^c^ | | | | |
| OCD symptom-dimensions | Hemisphere | Tract | AIC^a^ | |
|  |  |  | Linear regression models | Negative binomial models |
| Harm avoidance | Left | Dorsomedial PFC | **352.57** | 439.56 |
|  | Right | Dorsomedial PFC | **345.94** | 435.37 |
| Incompleteness | Left | Dorsomedial PFC | **361.87** | 448.36 |
| Extended sample 3 ^d^ | | | | |
| OCD symptom-dimensions | Hemisphere | Tract | AIC^a^ | |
|  |  |  | Linear regression models | Negative binomial models |
| Harm avoidance | Left | Dorsomedial PFC | **603.67** | 728.27 |
|  | Right | Dorsomedial PFC | **606.51** | 730.60 |
| Incompleteness | Left | Dorsomedial PFC | **602.97** | 721.87 |

Abbreviations: PFC, prefrontal cortex.

^a^ Lower AIC values reflect better fit. Best-fit metrics are reported in bold characters.

^b^ Extended sample 1 includes participants from sample 1 (healthy controls and obsessive-compulsive disorder) and sample 2 (obsessive-compulsive personality disorder).

^c^ Extended sample 2 includes participants from sample 1 (healthy controls and obsessive-compulsive disorder), sample 2 (obsessive-compulsive personality disorder), and sample 3 (non-OCD disorders).

^d^ Extended sample 3 includes participants from sample 1 (healthy controls and obsessive-compulsive disorder), sample 2 (obsessive-compulsive personality disorder), sample 3 (non-OCD disorders), and original sample (healthy controls and obsessive-compulsive disorder).

**Supplemental Table 5. Relationships between FA of PFC-striatum connections and OCD symptom-dimensions in the replication model (HC and OCD).**

| OCD dimension | Hemisphere | Tract | β | P | Q ^a^ |
| --- | --- | --- | --- | --- | --- |
| Harm avoidance | Left | Dorsolateral PFC | 0.15 | 0.167 | 0.825 |
|  |  | Dorsomedial PFC | 0.06 | 0.598 | 0.825 |
|  |  | Ventrolateral PFC | 0.19 | 0.114 | 0.825 |
|  |  | Ventromedial PFC | 0.05 | 0.670 | 0.825 |
|  | Right | Dorsolateral PFC | 0.08 | 0.498 | 0.825 |
|  |  | Dorsomedial PFC | 0.10 | 0.411 | 0.825 |
|  |  | Ventrolateral PFC | -0.07 | 0.561 | 0.825 |
|  |  | Ventromedial PFC | 0.05 | 0.645 | 0.825 |
| Incompleteness | Left | Dorsolateral PFC | 0.16 | 0.163 | 0.825 |
|  |  | Dorsomedial PFC | < 0.01 | 0.998 | 0.998 |
|  |  | Ventrolateral PFC | 0.13 | 0.299 | 0.825 |
|  |  | Ventromedial PFC | -0.01 | 0.932 | 0.994 |
|  | Right | Dorsolateral PFC | 0.07 | 0.562 | 0.825 |
|  |  | Dorsomedial PFC | 0.06 | 0.649 | 0.825 |
|  |  | Ventrolateral PFC | -0.11 | 0.342 | 0.825 |
|  |  | Ventromedial PFC | 0.01 | 0.896 | 0.994 |

Abbreviations: HC, Healthy controls; FA, Fractional Anisotropy; OCD, Obsessive-compulsive disorder.

^a^ Q represents the P-values after correction for multiple comparison.

**Supplemental Table 6. Association between OCD symptom dimensions and FA – Within group models.**

| Groups | OCD symptom-dimension | Tract | β | P | Q ^a^ |
| --- | --- | --- | --- | --- | --- |
| HC | Harm avoidance | Left dorsomedial PFC | 0.17 | 0.348 | 0.681 |
|  |  | Right dorsomedial PFC | 0.26 | 0.198 | 0.681 |
|  | Incompleteness | Left dorsomedial PFC | 0.23 | 0.309 | 0.680 |
| OCD | Harm avoidance | Left dorsomedial PFC | -0.08 | 0.569 | 0.854 |
|  |  | Right dorsomedial PFC | -0.06 | 0.702 | 0.936 |
|  | Incompleteness | Left dorsomedial PFC | -0.15 | 0.397 | 0.681 |
| OCPD | Harm avoidance | Left dorsomedial PFC | 0.05 | 0.884 | 0.964 |
|  |  | Right dorsomedial PFC | 0.08 | 0.797 | 0.956 |
|  | Incompleteness | Left dorsomedial PFC | 0.24 | 0.319 | 0.681 |
| Non-OCD | Harm avoidance | Left dorsomedial PFC | 0.01 | 0.966 | 0.966 |
|  |  | Right dorsomedial PFC | -0.24 | 0.373 | 0.681 |
|  | Incompleteness | Left dorsomedial PFC | -0.37 | 0.100 | 0.681 |

Abbreviations: FA, Fractional Anisotropy; HC, Healthy controls; OCD, Obsessive-compulsive disorder; OCPD, Obsessive-compulsive personality disorder.

^a^ Q represents the P-values after correction for multiple comparison.

**Supplemental Table 7. Extended models investigating non-significant relationships between FA of PFC-thalamus connections and OCD symptom-dimensions.**

| OCD symptom-dimensions | Hemisphere | Tract | Extended sample 1 ^a^ | | | Extended sample 2 ^b^ | | | Extended sample 3 ^c^ | | |
| --- | --- | --- | --- | --- | --- | --- | --- | --- | --- | --- | --- |
|  |  |  | β | P ^d^ | Q ^e^ | β | P ^d^ | Q ^e^ | β | P ^d^ | Q ^e^ |
| Harm avoidance | Left | Dorsolateral PFC | 0.10 | 0.383 | 0.982 | 0.07 | 0.465 | 0.991 | 0.05 | 0.514 | 0.980 |
|  |  | Ventrolateral PFC | -0.01 | 0.906 | 0.982 | -0.04 | 0.697 | 0.991 | < 0.01 | 0.979 | 0.980 |
|  |  | Ventromedial PFC | -0.02 | 0.860 | 0.982 | -0.02 | 0.792 | 0.991 | -0.06 | 0.367 | 0.980 |
|  | Right | Dorsolateral PFC | -0.06 | 0.584 | 0.982 | -0.08 | 0.412 | 0.991 | -0.04 | 0.569 | 0.980 |
|  |  | Ventrolateral PFC | 0.03 | 0.791 | 0.982 | < 0.01 | 0.991 | 0.991 | < 0.01 | 0.980 | 0.980 |
|  |  | Ventromedial PFC | -0.09 | 0.397 | 0.982 | -0.14 | 0.154 | 0.991 | -0.08 | 0.298 | 0.980 |
| Incompleteness | Left | Dorsolateral PFC | 0.04 | 0.744 | 0.982 | -0.01 | 0.903 | 0.991 | 0.04 | 0.576 | 0.980 |
|  |  | Ventrolateral PFC | 0.04 | 0.688 | 0.982 | < 0.01 | 0.969 | 0.991 | < 0.01 | 0.975 | 0.980 |
|  |  | Ventromedial PFC | < 0.01 | 0.998 | 0.998 | 0.02 | 0.821 | 0.991 | 0.01 | 0.855 | 0.980 |
|  | Right | Dorsolateral PFC | 0.03 | 0.780 | 0.982 | -0.05 | 0.636 | 0.991 | -0.04 | 0.601 | 0.980 |
|  |  | Dorsomedial PFC | 0.20 | **0.044** | 0.572 | 0.10 | 0.279 | 0.991 | 0.01 | 0.866 | 0.980 |
|  |  | Ventrolateral PFC | 0.07 | 0.503 | 0.982 | 0.02 | 0.859 | 0.991 | < 0.01 | 0.980 | 0.980 |
|  |  | Ventromedial PFC | -0.16 | 0.128 | 0.832 | -0.22 | **0.024** | 0.312 | -0.10 | 0.187 | 0.980 |

Abbreviations: FA, Fractional Anisotropy; PFC, Prefrontal cortex; OCD, Obsessive-compulsive disorder.

^a^ Extended sample1 includes participants from sample 1 (healthy controls and obsessive-compulsive disorder) and sample 2 (obsessive-compulsive personality disorder).

^b^ Extended sample 2 includes participants from sample 1 (healthy controls and obsessive-compulsive disorder), sample 2 (obsessive-compulsive personality disorder), and sample 3 (non-OCD disorders).

^c^ Extended sample 3 includes participants from sample 1 (healthy controls and obsessive-compulsive disorder), sample 2 (obsessive-compulsive personality disorder), sample 3 (non-OCD disorders), and original sample (healthy controls and obsessive-compulsive disorder).

^d^ P-values ≤ 0.05 are reported in bold characters.

^e^ Q represents the P-values after correction for multiple comparison.

**Supplemental Table 8. Relationships between RD and AD of PFC-thalamus connections and OCD symptom-dimensions in the replication model (HC and OCD).**

| OCD symptom-dimension | Hemisphere | Tract | RD | | | AD | | |
| --- | --- | --- | --- | --- | --- | --- | --- | --- |
|  |  |  | β | P ^a^ | Q ^a,b^ | β | P ^a^ | Q ^a,b^ |
| Harm avoidance | Left | Dorsolateral PFC | -0.14 | 0.241 | 0.551 | -0.08 | 0.478 | 0.637 |
|  |  | Dorsomedial PFC | -0.28 | **0.011** | 0.120 | 0.02 | 0.864 | 0.900 |
|  |  | Ventrolateral PFC | -0.04 | 0.738 | 0.787 | -0.15 | 0.218 | 0.575 |
|  |  | Ventromedial PFC | -0.07 | 0.501 | 0.618 | -0.09 | 0.395 | 0.575 |
|  | Right | Dorsolateral PFC | -0.04 | 0.669 | 0.765 | -0.15 | 0.161 | 0.575 |
|  |  | Dorsomedial PFC | -0.23 | **0.029** | 0.155 | 0.09 | 0.377 | 0.575 |
|  |  | Ventrolateral PFC | -0.13 | 0.233 | 0.551 | -0.11 | 0.308 | 0.575 |
|  |  | Ventromedial PFC | 0.01 | 0.911 | 0.911 | -0.03 | 0.806 | 0.900 |
| Incompleteness | Left | Dorsolateral PFC | -0.08 | 0.496 | 0.618 | -0.12 | 0.315 | 0.575 |
|  |  | Dorsomedial PFC | -0.27 | **0.015** | 0.120 | 0.03 | 0.757 | 0.900 |
|  |  | Ventrolateral PFC | -0.10 | 0.394 | 0.618 | -0.15 | 0.220 | 0.575 |
|  |  | Ventromedial PFC | -0.08 | 0.488 | 0.618 | -0.10 | 0.387 | 0.575 |
|  | Right | Dorsolateral PFC | -0.07 | 0.502 | 0.618 | -0.14 | 0.217 | 0.575 |
|  |  | Dorsomedial PFC | -0.21 | *0.058* | 0.232 | 0.12 | 0.288 | 0.575 |
|  |  | Ventrolateral PFC | -0.13 | 0.224 | 0.551 | -0.10 | 0.375 | 0.575 |
|  |  | Ventromedial PFC | 0.09 | 0.457 | 0.618 | 0.01 | 0.900 | 0.900 |

Abbreviations: RD, radial diffusivity; AD, axial diffusivity; PFC, Prefrontal cortex; OCD, Obsessive-compulsive disorder.

^a^ P-values ≤ 0.05 are reported in bold characters.

^b^ Q represents the P-values after correction for multiple comparison.

**Supplemental Table 9. Relationships between RD and AD of PFC-striatum connections and OCD symptom-dimensions in the replication model (HC and OCD).**

| OCD symptom-dimension | Hemisphere | Tract | RD | | | AD | | |
| --- | --- | --- | --- | --- | --- | --- | --- | --- |
|  |  |  | β | P | Q | β | P | Q |
| Harm  avoidance | Left | Dorsolateral PFC | 0.01 | 0.963 | 0.996 | 0.11 | 0.363 | 0.797 |
|  |  | Dorsomedial PFC | < 0.01 | 0.996 | 0.996 | 0.05 | 0.710 | 0.874 |
|  |  | Ventrolateral PFC | 0.10 | 0.320 | 0.718 | 0.19 | *0.082* | 0.797 |
|  |  | Ventromedial PFC | 0.17 | 0.142 | 0.718 | 0.15 | 0.186 | 0.797 |
|  | Right | Dorsolateral PFC | -0.07 | 0.497 | 0.884 | -0.03 | 0.812 | 0.928 |
|  |  | Dorsomedial PFC | 0.01 | 0.916 | 0.996 | 0.07 | 0.548 | 0.797 |
|  |  | Ventrolateral PFC | 0.16 | 0.135 | 0.718 | 0.09 | 0.421 | 0.797 |
|  |  | Ventromedial PFC | 0.10 | 0.359 | 0.718 | 0.09 | 0.376 | 0.797 |
| Incompleteness | Left | Dorsolateral PFC | -0.04 | 0.737 | 0.996 | 0.09 | 0.480 | 0.797 |
|  |  | Dorsomedial PFC | -0.02 | 0.861 | 0.996 | < 0.01 | 0.978 | 0.978 |
|  |  | Ventrolateral PFC | 0.11 | 0.297 | 0.718 | 0.18 | 0.120 | 0.797 |
|  |  | Ventromedial PFC | 0.14 | 0.226 | 0.718 | 0.12 | 0.324 | 0.797 |
|  | Right | Dorsolateral PFC | 0.02 | 0.856 | 0.996 | 0.05 | 0.702 | 0.874 |
|  |  | Dorsomedial PFC | -0.04 | 0.708 | 0.996 | 0.01 | 0.950 | 0.978 |
|  |  | Ventrolateral PFC | 0.15 | 0.160 | 0.718 | 0.07 | 0.544 | 0.797 |
|  |  | Ventromedial PFC | 0.12 | 0.270 | 0.718 | 0.10 | 0.346 | 0.797 |

Abbreviations: RD, radial diffusivity; AD, axial diffusivity; PFC, Prefrontal cortex; OCD, Obsessive-compulsive disorder.

**Supplemental Table 10. Effect of psychotropic mediations (Yes/No) on FA of cortical-subcortical connections of OCD participants.**

| Subcortical region of interest | Hemisphere | Tract | OCD participants in sample 1 | | | OCD participants in samples 1 and 4 | | |
| --- | --- | --- | --- | --- | --- | --- | --- | --- |
|  |  |  | F | P ^a^ | Q ^b^ | F | P ^a^ | Q ^b^ |
| Thalamus | Left | Dorsolateral PFC | 0.92 | 0.344 | 0.805 | 0.45 | 0.505 | 0.577 |
|  |  | Dorsomedial PFC | < 0.01 | 0.966 | 0.966 | 0.68 | 0.412 | 0.577 |
|  |  | Ventrolateral PFC | 1.28 | 0.266 | 0.805 | 0.51 | 0.479 | 0.577 |
|  |  | Ventromedial PFC | 0.16 | 0.695 | 0.805 | 5.01 | 0.028 | 0.224 |
|  | Right | Dorsolateral PFC | 0.43 | 0.514 | 0.805 | 1.87 | 0.175 | 0.566 |
|  |  | Dorsomedial PFC | 1.13 | 0.295 | 0.805 | 1.18 | 0.28 | 0.566 |
|  |  | Ventrolateral PFC | 0.15 | 0.704 | 0.805 | 1.17 | 0.283 | 0.566 |
|  |  | Ventromedial PFC | 0.27 | 0.607 | 0.805 | 0.07 | 0.799 | 0.799 |
| Striatum | Left | Dorsolateral PFC | 0.51 | 0.481 | 0.802 | 1.04 | 0.311 | 0.415 |
|  |  | Dorsomedial PFC | 0.03 | 0.858 | 0.867 | 0.07 | 0.797 | 0.797 |
|  |  | Ventrolateral PFC | 0.14 | 0.711 | 0.867 | 1.43 | 0.235 | 0.415 |
|  |  | Ventromedial PFC | 0.46 | 0.501 | 0.802 | 5.48 | **0.022** | 0.176 |
|  | Right | Dorsolateral PFC | 0.95 | 0.335 | 0.802 | 2.68 | 0.106 | 0.415 |
|  |  | Dorsomedial PFC | 0.03 | 0.867 | 0.867 | 0.07 | 0.793 | 0.797 |
|  |  | Ventrolateral PFC | 0.52 | 0.476 | 0.802 | 1.74 | 0.192 | 0.415 |
|  |  | Ventromedial PFC | 1.63 | 0.209 | 0.802 | 1.20 | 0.270 | 0.415 |

^a^ P-values ≤ 0.05 are reported in bold characters.

^b^ Q represents the P-values after correction for multiple comparison.

**Supplemental Table 11. Effect of illness duration on FA of cortical-subcortical connections of OCD participants.**

| Subcortical region of interest | Hemisphere | Tract | OCD participants in sample 1 | | | OCD participants in samples 1 and 4 | | |
| --- | --- | --- | --- | --- | --- | --- | --- | --- |
|  |  |  | β | P ^a^ | Q ^b^ | β | P ^a^ | Q ^b^ |
| Thalamus | Left | Dorsolateral PFC | -0.14 | 0.504 | 0.713 | -0.23 | *0.072* | 0.288 |
|  |  | Dorsomedial PFC | -0.04 | 0.869 | 0.869 | 0.04 | 0.777 | 0.777 |
|  |  | Ventrolateral PFC | -0.08 | 0.716 | 0.818 | -0.14 | 0.297 | 0.339 |
|  |  | Ventromedial PFC | 0.15 | 0.535 | 0.713 | -0.20 | 0.178 | 0.288 |
|  | Right | Dorsolateral PFC | -0.54 | **0.026** | 0.208 | -0.32 | **0.025** | 0.200 |
|  |  | Dorsomedial PFC | 0.21 | 0.428 | 0.713 | 0.21 | 0.156 | 0.288 |
|  |  | Ventrolateral PFC | -0.30 | 0.201 | 0.536 | -0.16 | 0.251 | 0.335 |
|  |  | Ventromedial PFC | -0.39 | *0.082* | 0.328 | -0.17 | 0.180 | 0.288 |
| Striatum | Left | Dorsolateral PFC | 0.19 | 0.378 | 0.853 | <0.01 | 0.997 | 0.997 |
|  |  | Dorsomedial PFC | 0.04 | 0.864 | 0.864 | -0.05 | 0.699 | 0.799 |
|  |  | Ventrolateral PFC | 0.04 | 0.863 | 0.864 | -0.12 | 0.398 | 0.757 |
|  |  | Ventromedial PFC | -0.12 | 0.640 | 0.853 | -0.24 | 0.076 | 0.608 |
|  | Right | Dorsolateral PFC | -0.20 | 0.374 | 0.853 | -0.15 | 0.230 | 0.757 |
|  |  | Dorsomedial PFC | 0.12 | 0.597 | 0.853 | -0.11 | 0.423 | 0.757 |
|  |  | Ventrolateral PFC | 0.20 | 0.424 | 0.853 | 0.05 | 0.668 | 0.799 |
|  |  | Ventromedial PFC | 0.14 | 0.524 | 0.853 | -0.09 | 0.473 | 0.757 |

^a^ P-values ≤ 0.05 are reported in bold characters; P values between 0.05 and 0.10 are reported in italics.

^b^ Q represents the P-values after correction for multiple comparison.

**Supplemental Table 12. Relationships between FA of SFG-thalamus and SFG-striatum connections and OCD symptom-dimensions in the replication model (HC and OCD).**

| Symptom dimension | Subcortical region | Cortical region | β | P ^a^ | Q ^b^ |
| --- | --- | --- | --- | --- | --- |
| Harm avoidance | Thalamus | Left SFG | 0.27 | **0.022** | *0.077* |
|  |  | Right SFG | 0.16 | 0.157 | 0.157 |
|  | Striatum | Left SFG | 0.21 | *0.073* | *0.099* |
|  |  | Right SFG | 0.2 | *0.074* | *0.099* |
| Incompleteness | Thalamus | Left SFG | 0.27 | **0.029** | *0.077* |
|  |  | Right SFG | 0.18 | 0.118 | 0.135 |
|  | Striatum | Left SFG | 0.27 | **0.028** | *0.077* |
|  |  | Right SFG | 0.22 | *0.061* | *0.099* |

^a^ P-values ≤ 0.05 are reported in bold characters; P values between 0.05 and 0.10 are reported in italics.

^b^ Q represents the P-values after correction for multiple comparison.

**Supplemental Table 13. Between-group differences in the number of streamlines.**

| Subcortical brain region | Hemisphere | Tract | F | P^a^ | Q^b^ |
| --- | --- | --- | --- | --- | --- |
| Thalamus | Left | Dorsolateral PFC | < 0.01 | 0.997 | 0.997 |
|  |  | Dorsomedial PFC | 0.11 | 0.895 | 0.997 |
|  |  | Ventrolateral PFC | 1.10 | 0.335 | 0.893 |
|  |  | Ventromedial PFC | 0.69 | 0.506 | 0.996 |
|  | Right | Dorsolateral PFC | 1.62 | 0.202 | 0.808 |
|  |  | Dorsomedial PFC ^c^ | 10.25 | **< 0.001** | **< 0.001** ^c^ |
|  |  | Ventrolateral PFC | 0.40 | 0.672 | 0.996 |
|  |  | Ventromedial PFC | 0.29 | 0.747 | 0.996 |
| Striatum | Left | Dorsolateral PFC | 1.08 | 0.343 | 0.932 |
|  |  | Dorsomedial PFC | 0.90 | 0.408 | 0.932 |
|  |  | Ventrolateral PFC | 0.22 | 0.804 | 0.967 |
|  |  | Ventromedial PFC | 0.12 | 0.888 | 0.967 |
|  | Right | Dorsolateral PFC | 0.83 | 0.438 | 0.932 |
|  |  | Dorsomedial PFC | 0.77 | 0.466 | 0.932 |
|  |  | Ventrolateral PFC | 0.10 | 0.907 | 0.967 |
|  |  | Ventromedial PFC | 0.03 | 0.967 | 0.967 |

^a^ P-values ≤ 0.05 are reported in bold characters.

^b^ Q represents the P-values after correction for multiple comparison.

^c^ There was no association between the number of streamlines and FA in the Right dorsomedial PFC connection (P>0.05).

**Supplemental Table 14. Between-group differences in FA in the PFC-thalamus connections.**

| Groups | Hemisphere | Tract | F | P ^a^ | Q ^a,b^ |
| --- | --- | --- | --- | --- | --- |
| HC versus OCD | Left | Dorsolateral PFC | 0.59 | 0.443 | 0.741 |
|  |  | Dorsomedial PFC | 6.09 | **0.016** | 0.128 |
|  |  | Ventrolateral PFC | 0.26 | 0.610 | 0.741 |
|  |  | Ventromedial PFC | 0.32 | 0.576 | 0.741 |
|  | Right | Dorsolateral PFC | 0.21 | 0.648 | 0.741 |
|  |  | Dorsomedial PFC | 2.93 | 0.091 | 0.364 |
|  |  | Ventrolateral PFC | 0.29 | 0.589 | 0.741 |
|  |  | Ventromedial PFC | 0.02 | 0.898 | 0.898 |
| HC versus OCPD | Left | Dorsolateral PFC | 1.56 | 0.217 | 0.868 |
|  |  | Dorsomedial PFC | 1.95 | 0.168 | 0.868 |
|  |  | Ventrolateral PFC | 0.16 | 0.690 | 0.957 |
|  |  | Ventromedial PFC | 0.06 | 0.807 | 0.957 |
|  | Right | Dorsolateral PFC | 0.21 | 0.646 | 0.957 |
|  |  | Dorsomedial PFC | 0.04 | 0.847 | 0.957 |
|  |  | Ventrolateral PFC | <0.01 | 0.957 | 0.957 |
|  |  | Ventromedial PFC | 0.09 | 0.772 | 0.957 |
| HC versus Non-OCD | Left | Dorsolateral PFC | <0.01 | 0.994 | 0.994 |
|  |  | Dorsomedial PFC | 0.69 | 0.409 | 0.918 |
|  |  | Ventrolateral PFC | 0.21 | 0.651 | 0.918 |
|  |  | Ventromedial PFC | 1.30 | 0.260 | 0.918 |
|  | Right | Dorsolateral PFC | 2.26 | 0.138 | 0.918 |
|  |  | Dorsomedial PFC | 0.07 | 0.798 | 0.918 |
|  |  | Ventrolateral PFC | 0.24 | 0.629 | 0.918 |
|  |  | Ventromedial PFC | 0.06 | 0.803 | 0.918 |
| HC versus All clinical groups (OCD, OCPD and Non-OCD) | Left | Dorsolateral PFC | 1.07 | 0.302 | 0.706 |
|  |  | Dorsomedial PFC | 5.42 | **0.021** | 0.168 |
|  |  | Ventrolateral PFC | 0.43 | 0.510 | 0.816 |
|  |  | Ventromedial PFC | 0.01 | 0.976 | 0.976 |
|  | Right | Dorsolateral PFC | 1.02 | 0.314 | 0.706 |
|  |  | Dorsomedial PFC | 0.86 | 0.353 | 0.706 |
|  |  | Ventrolateral PFC | 0.03 | 0.855 | 0.976 |
|  |  | Ventromedial PFC | 0.01 | 0.920 | 0.976 |
| OCD versus OCPD | Left | Dorsolateral PFC | 0.59 | 0.447 | 0.996 |
|  |  | Dorsomedial PFC | 0.45 | 0.505 | 0.996 |
|  |  | Ventrolateral PFC | <0.01 | 0.996 | 0.996 |
|  |  | Ventromedial PFC | 0.06 | 0.805 | 0.996 |
|  | Right | Dorsolateral PFC | 0.02 | 0.894 | 0.996 |
|  |  | Dorsomedial PFC | 2.53 | 0.116 | 0.928 |
|  |  | Ventrolateral PFC | 0.20 | 0.654 | 0.996 |
|  |  | Ventromedial PFC | 0.15 | 0.699 | 0.990 |
| OCD versus Non-OCD | Left | Dorsolateral PFC | 0.95 | 0.334 | 0.534 |
|  |  | Dorsomedial PFC | 0.57 | 0.453 | 0.604 |
|  |  | Ventrolateral PFC | 0.01 | 0.905 | 0.905 |
|  |  | Ventromedial PFC | 3.53 | 0.067 | 0.483 |
|  | Right | Dorsolateral PFC | 2.18 | 0.147 | 0.483 |
|  |  | Dorsomedial PFC | 1.34 | 0.253 | 0.506 |
|  |  | Ventrolateral PFC | 1.85 | 0.181 | 0.483 |
|  |  | Ventromedial PFC | 0.02 | 0.889 | 0.905 |
| OCPD versus Non-OCD | Left | Dorsolateral PFC | 1.46 | 0.234 | 0.931 |
|  |  | Dorsomedial PFC | 0.13 | 0.717 | >0.999 |
|  |  | Ventrolateral PFC | 0.01 | 0.946 | >0.999 |
|  |  | Ventromedial PFC | 2.55 | 0.118 | 0.931 |
|  | Right | Dorsolateral PFC | 0.90 | 0.349 | 0.931 |
|  |  | Dorsomedial PFC | <0.01 | 0.955 | >0.999 |
|  |  | Ventrolateral PFC | 0.34 | 0.565 | >0.999 |
|  |  | Ventromedial PFC | <0.01 | >0.999 | >0.999 |

Abbreviations: FA, Fractional Anisotropy; HC, Healthy controls; OCD, Obsessive-compulsive disorder; OCPD, Obsessive-compulsive personality disorder.

^a^ P-values ≤ 0.05 are reported in bold characters.

^b^ Q represents the P-values after correction for multiple comparison.

**Supplemental Table 15. Between-group differences in FA in the PFC-striatum connections.**

| Groups | Hemisphere | Tract | F | P | Q ^a^ |
| --- | --- | --- | --- | --- | --- |
| HC versus OCD | Left | Dorsolateral PFC | 0.07 | 0.793 | 0.906 |
|  |  | Dorsomedial PFC | 0.46 | 0.502 | 0.906 |
|  |  | Ventrolateral PFC | 0.14 | 0.706 | 0.906 |
|  |  | Ventromedial PFC | 1.13 | 0.292 | 0.779 |
|  | Right | Dorsolateral PFC | >0.01 | 0.954 | 0.954 |
|  |  | Dorsomedial PFC | 2.02 | 0.159 | 0.636 |
|  |  | Ventrolateral PFC | 2.59 | 0.111 | 0.636 |
|  |  | Ventromedial PFC | 0.17 | 0.679 | 0.906 |
| HC versus OCPD | Left | Dorsolateral PFC | 0.57 | 0.452 | 0.853 |
|  |  | Dorsomedial PFC | 2.66 | 0.109 | 0.675 |
|  |  | Ventrolateral PFC | 0.09 | 0.768 | 0.878 |
|  |  | Ventromedial PFC | 1.39 | 0.244 | 0.675 |
|  | Right | Dorsolateral PFC | 1.33 | 0.253 | 0.675 |
|  |  | Dorsomedial PFC | 0.22 | 0.640 | 0.853 |
|  |  | Ventrolateral PFC | <0.01 | 0.972 | 0.972 |
|  |  | Ventromedial PFC | 0.38 | 0.538 | 0.853 |
| HC versus Non-OCD | Left | Dorsolateral PFC | 0.09 | 0.768 | 0.851 |
|  |  | Dorsomedial PFC | 0.04 | 0.851 | 0.851 |
|  |  | Ventrolateral PFC | 0.20 | 0.654 | 0.851 |
|  |  | Ventromedial PFC | 0.57 | 0.455 | 0.851 |
|  | Right | Dorsolateral PFC | 0.09 | 0.771 | 0.851 |
|  |  | Dorsomedial PFC | 1.82 | 0.182 | 0.627 |
|  |  | Ventrolateral PFC | 1.44 | 0.235 | 0.627 |
|  |  | Ventromedial PFC | 1.65 | 0.204 | 0.627 |
| HC versus All clinical groups (OCD, OCPD and Non-OCD) | Left | Dorsolateral PFC | 0.29 | 0.593 | 0.863 |
|  |  | Dorsomedial PFC | <0.01 | 0.972 | 0.972 |
|  |  | Ventrolateral PFC | 0.26 | 0.613 | 0.863 |
|  |  | Ventromedial PFC | 0.23 | 0.630 | 0.863 |
|  | Right | Dorsolateral PFC | 0.10 | 0.755 | 0.863 |
|  |  | Dorsomedial PFC | 2.20 | 0.141 | 0.672 |
|  |  | Ventrolateral PFC | 1.92 | 0.168 | 0.672 |
|  |  | Ventromedial PFC | 0.20 | 0.658 | 0.863 |
| OCD versus OCPD | Left | Dorsolateral PFC | 0.36 | 0.551 | 0.630 |
|  |  | Dorsomedial PFC | 4.41 | 0.040 | 0.160 |
|  |  | Ventrolateral PFC | 0.00 | 0.961 | 0.961 |
|  |  | Ventromedial PFC | 4.93 | 0.030 | 0.160 |
|  | Right | Dorsolateral PFC | 1.15 | 0.287 | 0.507 |
|  |  | Dorsomedial PFC | 0.41 | 0.522 | 0.630 |
|  |  | Ventrolateral PFC | 1.72 | 0.194 | 0.507 |
|  |  | Ventromedial PFC | 1.02 | 0.317 | 0.507 |
| OCD versus Non-OCD | Left | Dorsolateral PFC | 0.22 | 0.640 | 0.923 |
|  |  | Dorsomedial PFC | 0.07 | 0.797 | 0.923 |
|  |  | Ventrolateral PFC | 0.05 | 0.826 | 0.923 |
|  |  | Ventromedial PFC | 0.03 | 0.854 | 0.923 |
|  | Right | Dorsolateral PFC | 0.32 | 0.574 | 0.923 |
|  |  | Dorsomedial PFC | 0.19 | 0.668 | 0.923 |
|  |  | Ventrolateral PFC | 0.01 | 0.923 | 0.923 |
|  |  | Ventromedial PFC | 1.19 | 0.280 | 0.923 |
| OCPD versus Non-OCD | Left | Dorsolateral PFC | 0.16 | 0.687 | 0.785 |
|  |  | Dorsomedial PFC | 3.10 | *0.086* | 0.251 |
|  |  | Ventrolateral PFC | 0.03 | 0.872 | 0.872 |
|  |  | Ventromedial PFC | 3.23 | *0.080* | 0.251 |
|  | Right | Dorsolateral PFC | 1.45 | 0.236 | 0.472 |
|  |  | Dorsomedial PFC | 0.52 | 0.476 | 0.635 |
|  |  | Ventrolateral PFC | 1.08 | 0.304 | 0.486 |
|  |  | Ventromedial PFC | 2.95 | *0.094* | 0.251 |

Abbreviations: FA, Fractional Anisotropy; HC, Healthy controls; OCD, Obsessive-compulsive disorder; OCPD, Obsessive-compulsive personality disorder.

^a^ Q represents the P-values after correction for multiple comparison.

**Supplemental Table 16. Association between FA and depressive symptoms.**

| Groups | Hemisphere | Tract | β | P | Q |
| --- | --- | --- | --- | --- | --- |
| Thalamus | Left | Dorsolateral PFC | 0.02 | 0.865 | 0.923 |
|  |  | Dorsomedial PFC | 0.08 | 0.379 | 0.923 |
|  |  | Ventrolateral PFC | -0.10 | 0.251 | 0.923 |
|  |  | Ventromedial PFC | -0.02 | 0.819 | 0.923 |
|  | Right | Dorsolateral PFC | -0.03 | 0.713 | 0.923 |
|  |  | Dorsomedial PFC | 0.06 | 0.507 | 0.923 |
|  |  | Ventrolateral PFC | 0.02 | 0.865 | 0.923 |
|  |  | Ventromedial PFC | -0.05 | 0.629 | 0.923 |
| Striatum | Left | Dorsolateral PFC | 0.05 | 0.611 | 0.923 |
|  |  | Dorsomedial PFC | <0.01 | 0.980 | 0.980 |
|  |  | Ventrolateral PFC | 0.04 | 0.697 | 0.923 |
|  |  | Ventromedial PFC | -0.05 | 0.556 | 0.923 |
|  | Right | Dorsolateral PFC | -0.02 | 0.840 | 0.923 |
|  |  | Dorsomedial PFC | -0.12 | 0.177 | 0.923 |
|  |  | Ventrolateral PFC | -0.12 | 0.177 | 0.923 |
|  |  | Ventromedial PFC | -0.07 | 0.468 | 0.923 |

Abbreviations: PFC – prefrontal cortex.

**SUPPLEMENTAL FIGURES.**

**Supplemental Figure 1. Relationship between FA of the right dorsomedial PFC-thalamus and harm avoidance in the current sample.**
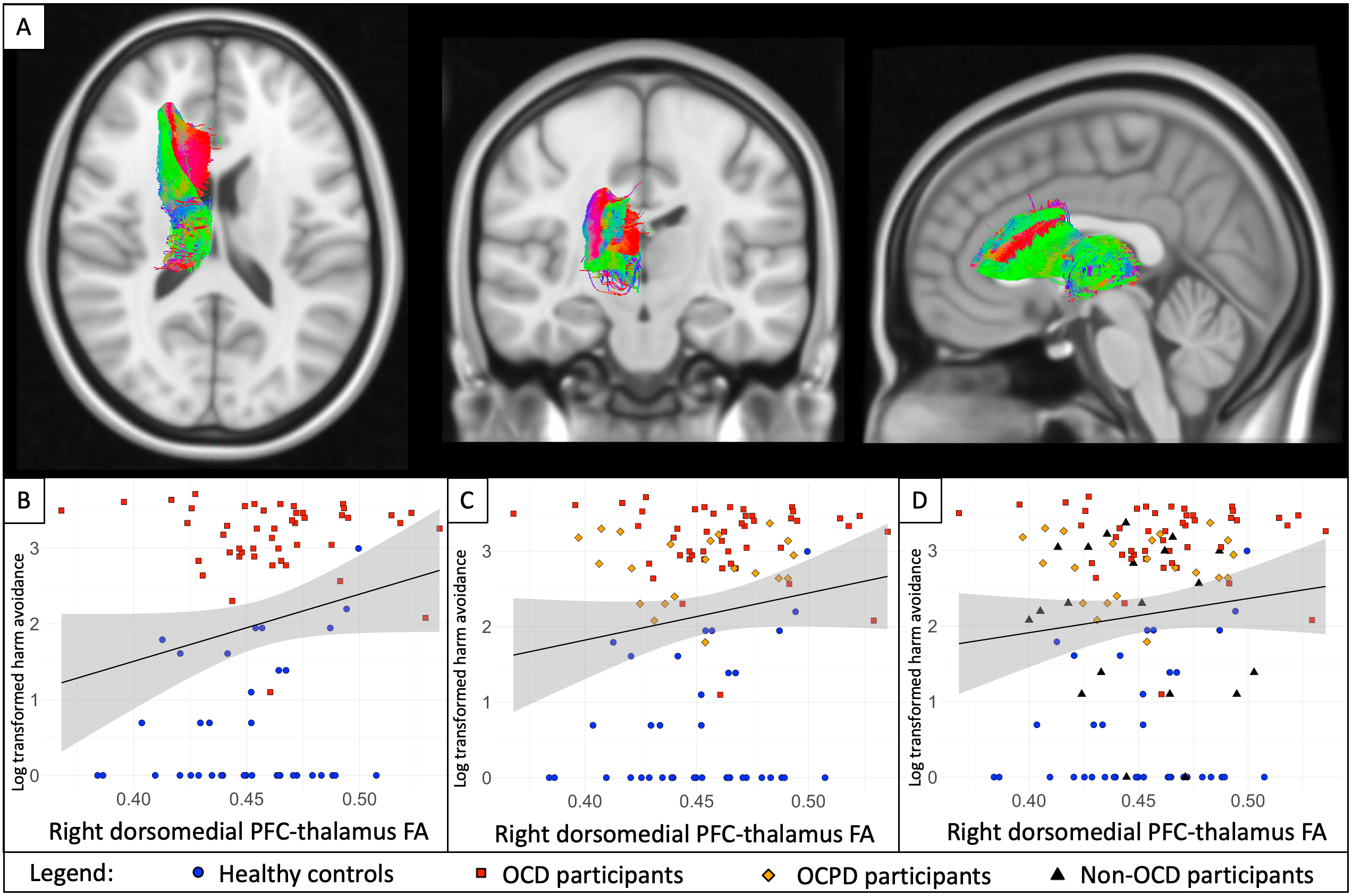


**Supplemental Figure 1 legend:** Panel A shows the reconstructed connection between right dorsomedial PFC and thalamus. Axial, coronal, and sagittal slices are shown. Colors indicate streamline orientation: green (anterior-posterior), blue (superior-inferior), and red (right-left). In this panel, the background is the standard Montreal Neurological Institute 152 space 1-mm brain. In Panels B-D, the x-axis shows the right dorsomedial PFC-thalamus FA and the y-axis shows the log transformed harm avoidance. Log transformation was performed as log(score + 1) to include participants with a score of 0 and avoid producing negative infinity. In Panel B, healthy controls and OCD participants are displayed. In Panel C, healthy controls, OCD, and OCPD participants are displayed. In Panel D, healthy controls, OCD, OCPD, and non-OCD participants are displayed. In all panels, the gray area represents 95% confidence intervals and the black line represents the regression line. Abbreviations: FA, Fractional anisotropy; PFC, Prefrontal cortex; OCD, Obsessive-compulsive disorder; OCPD, Obsessive-Compulsive Personality Disorder.

**Supplemental Figure 2. Relationship between FA of the left dorsomedial PFC-thalamus and incompleteness in the current sample.**


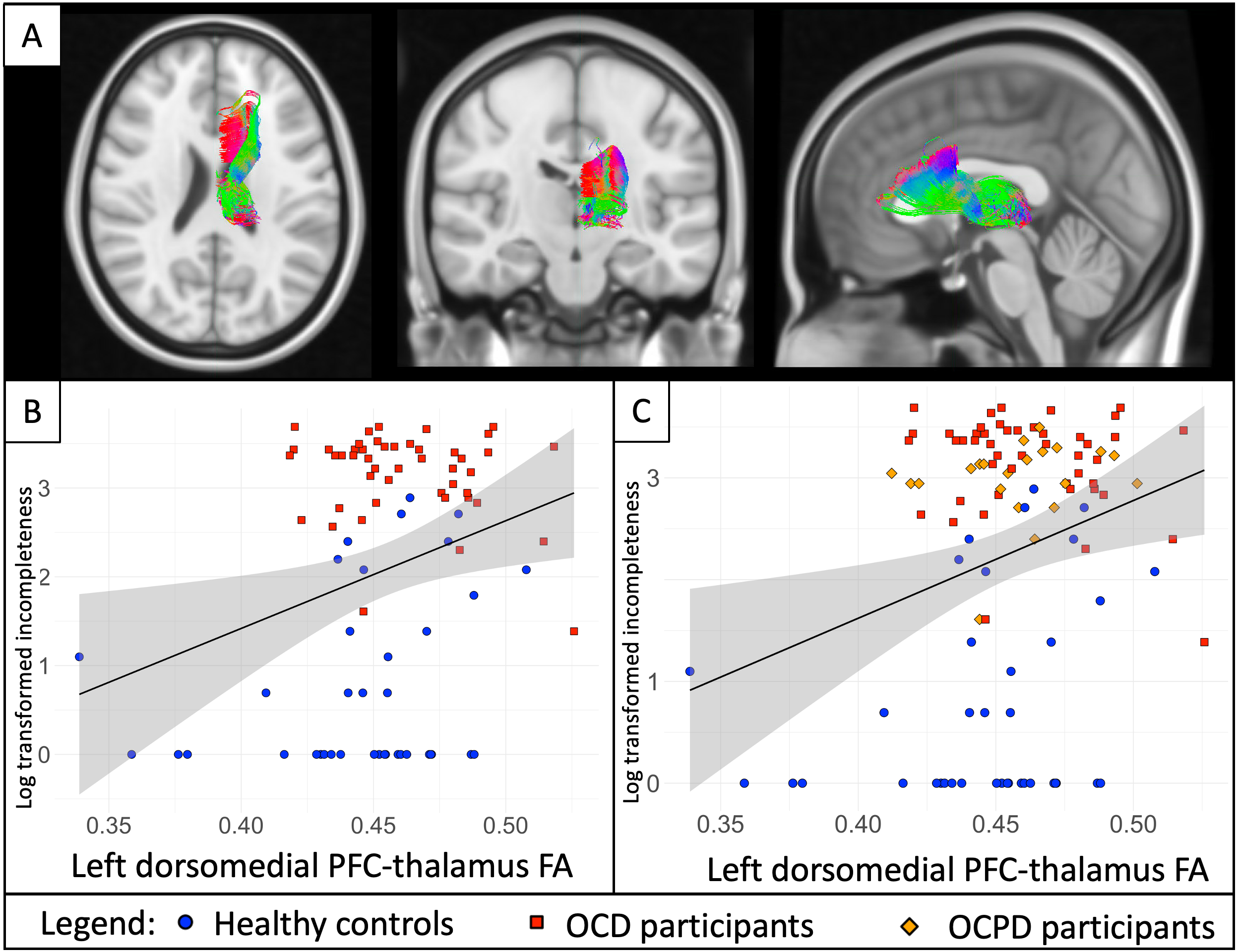


**Supplemental Figure 2 legend:** Panel A shows the reconstructed connection between left dorsomedial PFC and thalamus. Axial, coronal, and sagittal slices are shown. Colors indicate streamline orientation: green (anterior-posterior), blue (superior-inferior), and red (right-left). In this panel, the background is the standard Montreal Neurological Institute 152 space 1-mm brain. In Panels B and C, the x-axis shows the left dorsomedial PFC-thalamus FA and the y-axis shows the log transformed incompleteness. Log transformation was performed as log(score + 1) to include participants with a score of 0 and avoid producing negative infinity. In Panel B, healthy controls and OCD participants are displayed. In Panel C, healthy controls, OCD, and OCPD participants are displayed. In all panels, the gray area represents 95% confidence intervals and the black line represents the regression line. Abbreviations: FA, Fractional anisotropy; PFC, Prefrontal cortex; OCD, Obsessive-compulsive disorder; OCPD, Obsessive-Compulsive Personality Disorder.

**Supplemental Figure 3. Relationship between FA of the left dorsomedial PFC-thalamus and incompleteness in the model combining current and original samples.**

**
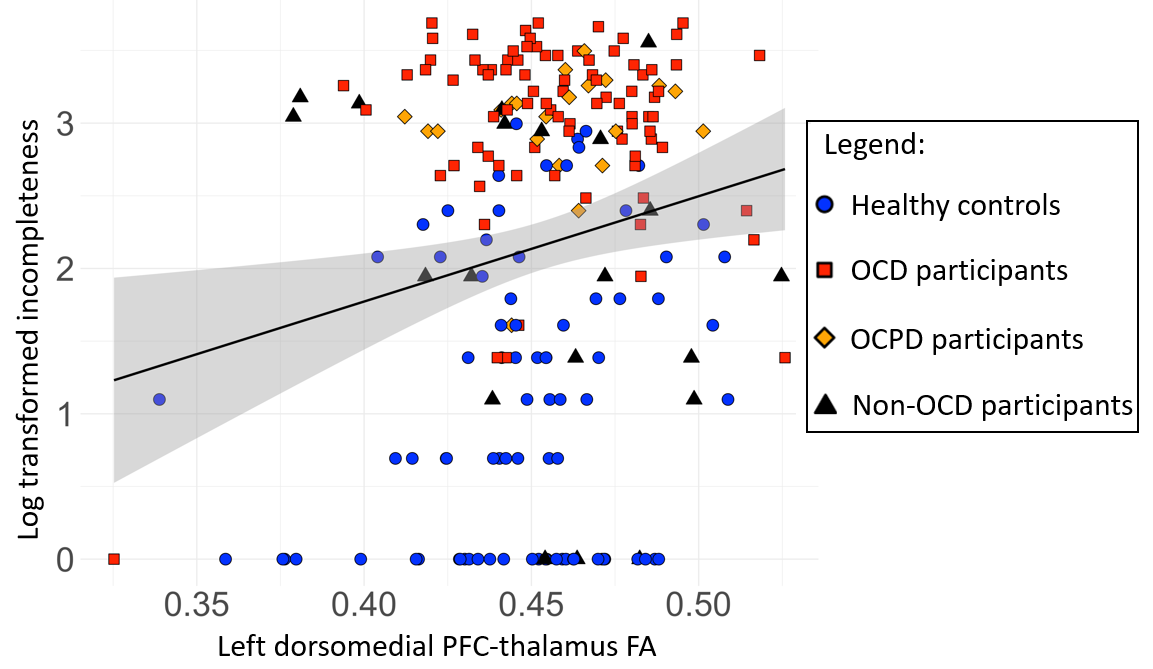
**

**Supplemental Figure 3 legend:** The x-axis shows the left dorsomedial PFC-thalamus FA and the y-axis shows the log transformed incompleteness. Log transformation was performed as log(score + 1) to include participants with a score of 0 and avoid producing negative infinity. In this scatter plot, healthy controls, OCD, OCPD, and non-OCD participants are displayed. Healthy controls and OCD participants include the current and original samples. The gray area represents 95% confidence interval, and the black line represents the regression line. Abbreviations: FA, Fractional anisotropy; PFC, Prefrontal cortex; OCD, Obsessive-compulsive disorder; OCPD, Obsessive-Compulsive Personality Disorder.

**SUPPLEMENTAL REFERENCES**

1 Brown TA, Barlow DH. Anxiety and related disorders interview schedule for DSM-5 (ADIS-5)-adult and lifetime version: Clinician manual*.* Oxford University Press; 2014.

2 First M, Williams J, Benjamin L, Spitzer R. Structured clinical interview for DSM-5 personality disorders: SCID-5-PD. Testkatalog. 2016:30.

3 Tolin DF, Frost RO, Steketee G. A brief interview for assessing compulsive hoarding: the Hoarding Rating Scale-Interview. Psychiatry research. 2010;178(1):147–52.

4 Lima Santos JP, Versace A, Arora M, Bertocci MA, Chase HW, Skeba A, et al. Examining relationships among NODDI indices of white matter structure in prefrontal cortical-thalamic-striatal circuitry and OCD symptomatology. Translational Psychiatry. 2024;14(1):410.

5 Caruyer E, Lenglet C, Sapiro G, Deriche R. Design of multishell sampling schemes with uniform coverage in diffusion MRI. Magnetic resonance in medicine : official journal of the Society of Magnetic Resonance in Medicine / Society of Magnetic Resonance in Medicine. 2013;69(6):1534–40.

6 Smith SM, Jenkinson M, Woolrich MW, Beckmann CF, Behrens TE, Johansen-Berg H, et al. Advances in functional and structural MR image analysis and implementation as FSL. Neuroimage. 2004;23:S208–S19.

7 Andersson JL, Skare S, Ashburner J. How to correct susceptibility distortions in spin-echo echo-planar images: application to diffusion tensor imaging. Neuroimage. 2003;20(2):870–88.

8 Casey BJ, Cannonier T, Conley MI, Cohen AO, Barch DM, Heitzeg MM, et al. The adolescent brain cognitive development (ABCD) study: imaging acquisition across 21 sites. Developmental cognitive neuroscience. 2018;32:43–54.

9 Hagler Jr DJ, Hatton S, Cornejo MD, Makowski C, Fair DA, Dick AS, et al. Image processing and analysis methods for the Adolescent Brain Cognitive Development Study. Neuroimage. 2019;202:116091.
